# Potential Protective Effects of Pungent Flavor Components in Neurodegenerative Diseases

**DOI:** 10.3390/molecules29235700

**Published:** 2024-12-02

**Authors:** Fangxin Guo, Xudi Qin, Jian Mao, Yan Xu, Jianping Xie

**Affiliations:** 1 Beijing Life Science Academy (BLSA), Beijing 102209, China; 2 School of Basic Medical Sciences, College of Medicine, Zhengzhou University, Zhengzhou 450001, China; 3 Flavour Science Research Center, College of Chemistry, Zhengzhou University, Zhengzhou 450001, China

**Keywords:** neurodegenerative diseases, pungent flavor components, plant extracts, neuroprotective effects

## Abstract

Neurodegenerative diseases such as Alzheimer’s disease (AD), Parkinson’s disease (PD), and Huntington’s disease (HD) have become a major global health burden, but the detailed pathogeneses of neurodegenerative diseases are still unknown, and current treatments are mainly aimed at controlling symptoms; there are no curative treatments for neurodegenerative diseases or treatments for the progressive cognitive, behavioral, and functional impairments that they cause. Studies have shown that some plant extracts with pungent flavor components have a certain neuroprotective effect in neurodegenerative diseases, and their mechanisms mainly involve inhibiting neuronal apoptosis, promoting neuronal regeneration, reducing mitochondrial degeneration, and reducing the production of oxides such as reactive oxygen species in cells, which are of great significance for exploring the treatment of neurodegenerative diseases. In this review, we searched the PubMed database for relevant literature collected in the past 15 years. Finally, we summarized the protective effects of pungent flavor components such as capsaicin, piperine, curcumin, cannabinoids, allicin, and nicotine on the nervous system, focusing on the molecular mechanisms and signaling pathways that they activate. In addition, we also compiled and summarized the laboratory experiments, preclinical experiments, and effects of various pungent flavor components in neurodegenerative diseases. The goal is to further explore their potential as effective drugs for the treatment of neurodegenerative diseases and provide new ideas for further research on the specific protective mechanisms of these substances for the treatment of neurodegenerative diseases and the targets of drug action in the future.

## 1. Introduction

Neurodegenerative diseases are neurological disorders caused by the loss of neurons and/or their myelin sheaths, which results in a series of neurological dysfunctions over time, including Alzheimer’s disease (AD), Parkinson’s disease (PD), Huntington’s disease (HD), and amyotrophic lateral sclerosis (ALS) [1]. Neurodegenerative disease is characterized by progressive dysfunction and neuronal loss, with different disease systems involved and closely associated with the corresponding clinical manifestations [2]. There is a plethora of research on treatment modalities for neurodegenerative diseases, but there are no curative treatments for these diseases or the progressive cognitive, behavioral, and functional deficits that they cause [3].

Alzheimer’s disease (AD) is the most prevalent neurodegenerative disease in the world and the most common cause of dementia [2]. Early symptoms of AD may be present in about one in ten elder adults in developed countries, with a huge impact on patients’ later life indices [3]. There are two main pathological changes in AD: the accumulation of amyloid composed of β-amyloid (Aβ) outside neuronal cells, and the accumulation of neurofibrillary tangles (NFTs) formed through the aggregation of hyperphosphorylated tau proteins in neurons [4]. These two pathological changes are closely related to mitochondrial dysfunction and glial cell activation. In terms of clinical application, researchers have proposed amyloid, tau protein, neurodegeneration, and A/T/N classification and staging systems based on the imaging of protein lesions, cerebrospinal fluid (CSF) biomarkers, and structural imaging [5]. CSF and positron emission tomography (PET) biomarkers, in combination with current newer clinical criteria, could help to improve the in vivo diagnosis of patients [2]. The clinical criteria for AD have been described in the literature [5]. The approved treatments for AD include two cholinesterase inhibitors and memantine [6]. The former can maintain the levels of the neurotransmitter acetylcholine by inhibiting the enzymes implicated in its degradation, while the latter can inhibit glutamate release and promote the regeneration and repair of cholinergic neurons [7,8]. However, they only slow the progression of neurodegeneration; drugs that can prevent and cure AD or more efficiently slow its progression are yet to be found.

PD is the second-most common neurodegenerative disease, after AD, and is typified by its motor symptoms, known as resting tremor, rigidity, bradykinesia, and postural instability (TRAP); it negatively affects patients’ quality of life and incurs high healthcare costs [9,10]. PD has an extremely long clinical latency period, known as pre-PD. During this period, a series of preclinical symptoms of PD may provide clues for early diagnosis [9]. The main pathological feature of PD is the aggregation of α-synuclein (α-syn) in the hippocampus and corpus striatum, which promotes neuronal apoptosis and induces microglial polarization, among other effects. Based on large autopsy-confirmed cases, it is known that the clinical diagnostic accuracy for PD is only 80%, while the pathological diagnostic rate is somewhat higher [11]. There is also a lack of known biomarkers for PD; the diagnostic rate will be greatly improved by the discovery of specific biomarkers, and in the future it should be possible to recognize PD in the prodromal phase and implement neuroprotective interventions before the onset of motor symptoms [5]. The only primary prevention measure for PD to date has been the promotion of physical activity [9]. Although PD is currently incurable, relevant treatments can alleviate symptoms and improve quality of life. Currently, the mainstream therapeutic drug for PD is levodopa (L-DOPA), which can cross the blood–brain barrier and functions as a dopamine replacement, compensating for the loss of dopaminergic neurons in PD, but it cannot stop the progression of the disease [12,13].

HD, the result of a mutation in the *huntingtin* (HTT) gene, is the only purely genetic neurodegenerative disease, although there are other hereditary neurodegenerative diseases like spinocerebellar ataxia (SCA) [14,15]. It is characterized by motor abnormalities (most commonly chorea), psychiatric symptoms, and cognitive deficits [16,17]. It is caused by a pathogenic amplification (≥36 repeats) of the trinucleotide repeat cytosine–adenine–guanine (CAG) in the *huntingtin* (HTT) gene located on chromosome 4 [16]. The pathological features of HD are corpus striatum and neuronal deficits in the cerebral cortex, which are associated with the aggregation of toxic mutant Huntington’s proteins, but the exact mechanism is unclear [17,18,19]. Buprenazine and deuterobuprenazine effectively relieve the choreiform symptoms and are the only drugs approved by the FDA for the treatment of HD [16]. Selective serotonin reuptake inhibitors/norepinephrine reuptake inhibitors (SSRIs/SNRIs) and benzodiazepines, on the other hand, are used to treat the psychiatric symptoms of HD, and the dementia associated with HD can be treated with acetylcholinesterase inhibitors [16]. Current drugs can alleviate the symptoms of HD but cannot cure the neurodegeneration, so further research on the underlying pathological mechanisms is necessary [14]. New evidence has indicated that dietary fiber interventions may have therapeutic potential in HD, delaying clinical onset, and have implications for related disorders exhibiting dysfunction of the gut–brain axis, showing promise for HD treatment [20].

Although researchers have made great efforts to determine the pathogenesis of neurodegenerative disease, little is known about the pathogeneses of specific neurodegenerative diseases, and no method has been found that can completely cure a specific type of neurodegenerative disease. However, it has been found that some plant extracts with special pungent odors have certain neuroprotective effects in neurodegenerative diseases, and their mechanisms mainly involve inhibiting neuronal apoptosis, promoting neuronal regeneration to reduce mitochondrial degeneration and inactivation, and decreasing the production of oxidants such as reactive oxygen species in cells, which may offer new hope for protection against these diseases. For this review, we searched the PubMed database for relevant literature collected in the past 15 years. After reviewing the literature and excluding studies with content not closely related to the article’s theme, as well as those of poor quality, we ultimately included 195 articles from 2010 to 2024 for summarization and synthesis. Therefore, in this review, we focus on the mechanisms of the neuroprotective effects of stimulating flavor components, such as capsaicin, piperine, curcumin, cannabinoids, and allicin. We also discuss the agonism of TRVP1 by capsaicin and the modulatory effects of CUR on the Wnt/β-catenin pathway and phosphatidylinositol-3 kinase/protein kinase B signaling pathway (PI3K/AKT signaling pathway), among other aspects. Furthermore, we discuss the inhibitory effects of piperine on AKT–mTORC1 signaling, the modulatory effects of cannabinoids on the PI3K/AKT/mTORC 1/BDNF and PPARγ pathways, and the activating effects of nicotine on the α7-nAChR/Erk 1/2 and Wnt/β-catenin pathways [21,22,23,24,25,26,27,28]. We also discuss the effects of capsaicin, piperine, and other stimulating flavor components, organize laboratory and preclinical experiments related to neurodegenerative diseases (AD, PD, and HD), and comprehensively explore the synergistic effects of these stimulating flavor components, providing new ideas for further investigations into their mechanisms.

## 2. Capsaicin

Capsaicin (trans-8-methyl-N-vanilly-6-nonenamide, CAP) is a vanillic acid derivative that is the main source of spicy flavor in plants of the genus *Capsicum* [29,30]. CAP consists of a vanillin group (head), amide group (neck), and fatty acid chain (tail) [31]. CAP’s activities are categorized into receptor-dependent and non-receptor-dependent pathways, but it mainly protects against neurodegeneration through transient receptor potential vanilloid 1 (TRPV1) [32]. Mainly through TRPV1, CAP regulates body temperature; increases blood flow and energy expenditure; reduces oxidative stress; alleviates pain and cognitive impairment; ameliorates ischemia, excitotoxicity, and hepatic failure-induced brain damage; and protects against atherosclerosis, cardiovascular disease, stroke, obesity, hypertension, cancer, and gastrointestinal and inflammatory diseases [30,33,34,35].

**Figure 1:**
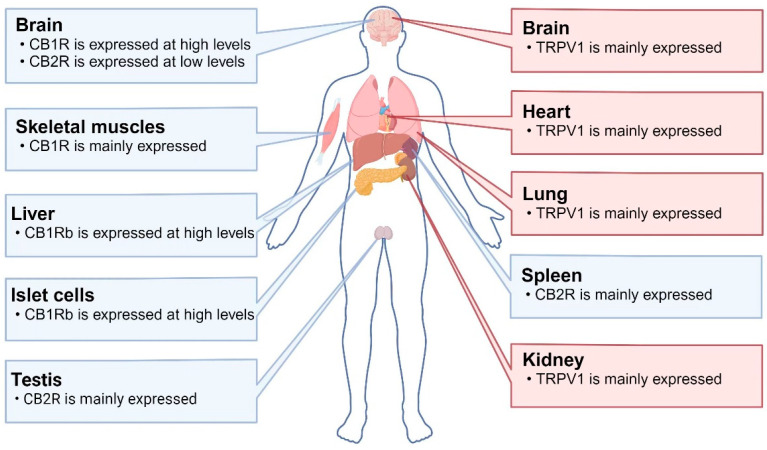
Distributions of TRPV1 and CBR in humans: CB1R is highly expressed in the brain and skeletal muscles. CB1Rb (a variant lacking 33 amino acids at the N-terminus) is highly expressed in the liver and islet cells. CB2R is mainly expressed in the testes and spleen, while it is expressed at low levels in the brain. TRPV1 is mainly expressed in the brain, heart, lungs, and kidneys. Abbreviations: CB1R: cannabinoid receptor 1; CB2R: cannabinoid receptor 2; TRPV1: transient receptor potential vanilloid 1.

TRPV1 channels are polymodal cation channels that allows cations to pass through, especially Ca^2+^ [36]. TRPV1 channels are mainly expressed in sensory neurons of the peripheral nervous system, and in the human CNS they are mainly distributed in the cerebral cortex, corpus striatum, and hippocampus, where they play roles in regulating cellular autophagy, synaptic transmission and plasticity, and cognitive adjustments [21,37,38] (Figure 1). TRPV1’s transmembrane core region contains six transmembrane helices (S1 to S6) per subunit and exhibits the same topology and other structural features as voltage-gated potassium channels, for which CAP is highly selective [31]. Upon the activation of TRVP1 channels by CAP, Ca^2+^ ions enter the cell and stimulate intracellular cascade reactions, including the control of regulatory T cells in the gut and activation of neuroimmune interactions [39,40,41].

**Figure 2:**
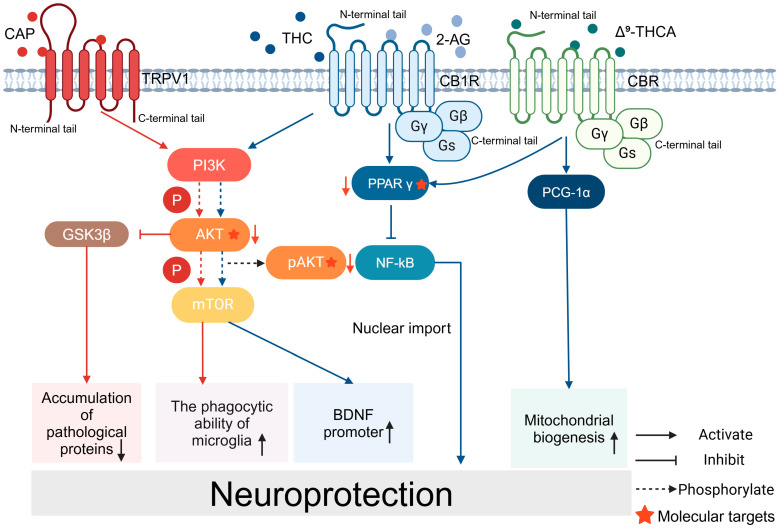
Signaling pathways for the neuroprotective effects of CAP and CB: CAP acts on TRPV1 and activates the PI3K/AKT/mTOR signaling pathway, enhances the phagocytosis of microglia, and activates the promoter of BDNF. CAP reduces the accumulation of pathological proteins through PI3K/AKT/GSK3β. Cannabinoids and their related products bind to CBR and exert neuroprotective effects. THC binds to CB1R and activates the PPARγ/NF-kB pathway. Δ^9^-THCA promotes mitochondrial biogenesis.

CAP has been shown to protect against a variety of neurodegenerative diseases. During the course of AD, CAP can reduce Aβ formation through several mechanisms, including promoting the maturation of deintegrin and metalloproteinase 10 (ADAM 10), shifting the direction of amyloid precursor protein (APP) cleavage and blocking the formation of Aβm, and ameliorating Aβ pathology through AKT/GSK-3β-mediated Nrf2 activation, which achieves protective effects [42,43]. Studies have shown that CAP inhibits the endocytosis of GluA2-containing α-amino-3-hydroxy-5-methyl-4-isoxazolepropionic acid receptor (AMPAR) through the activation of TRPV1 channels and effectively ameliorates spatial learning memory deficits and synaptic plasticity impairments in AD mice [44,45]. In addition, the activation of microglia, which exhibit M1 and M2 phenotypes, plays an important role in AD’s pathology; CAP regulates microglial activation by inhibiting AKT/mTOR pathway activity and reduces neuronal degeneration, reversing spatial memory deficits [21,42,43,44,46,47] (Figure 2). In addition, another group conducted a questionnaire survey to explore the protective effects of CAP. After conducting their survey on the dietary habits and cognitive statuses of 338 middle-aged and elderly people, it was found that the long-term use of CAP helped to enhance the cognitive function of middle-aged and elderly AD patients, which suggests that CAP could be a potential drug for the prevention and treatment of AD [35] (Table 1).

In PD, CAP treatment reduced motor dysfunction in a murine model of PD [48,49] In terms of pathology, CAP prevents the degeneration of dopaminergic neurons in the substantia nigra, increases the survival of DA neurons in the substantia nigra, and elevates DA levels in the corpus striatum [48,49,50,51]. Other studies have shown that the activation of the TRPV1 receptor by CAP enhanced the phagocytosis of microglia, reduced oxidative stress, and decreased the phosphorylation of α-syn, thereby promoting the survival of degenerate neurons, suggesting that the modulation of microglial metabolism by CAP could be a novel approach for the treatment of PD [52,53]. This suggests that the regulation of microglial metabolism with CAP may be a novel approach for treating PD [54,55]. In addition, astrocytes protect DA neurons from neurotoxicity. CAP stimulates TRPV1-ribosomal protein 70S6 kinase (TRPV 1-p70S6K) in astrocytes, which activates the p70S6K signaling pathway and promotes the production of endogenous ciliary neurotrophic factor (CNTF), preventing the persistent degeneration of DA in the nigral corpus striatum of rats, which alleviated motor deficits in a rat model of PD. The above studies demonstrated the neuroprotective potential of CAP to regulate the normalization of astrocyte functions [54,55]. CAP can also protect against PD through other pathways: in a 6-OHDA-induced cell model of PD, CAP reduced apoptosis by regulating actin gamma 1 (Actg 1) and glutathione S-transferase 2 (Gstα 2) [34]. The neuroprotective effect of CAP was also demonstrated in a *Drosophila* model of PD [56] (Table 1).

CAP is equally protective against other neurodegenerative diseases. Regarding the role of CAP in HD, early studies have shown that CAP significantly attenuates 3-nitropropionic acid-induced hypermotility and corpus striatum atrophy in HD rats [57,58,59]. In a crossover randomized controlled trial, patients with ALS showed significant relief of their ALS-related dysphagia signs after the administration of CAP [60] (Table 1).

In conclusion, CAP alleviates the pathological changes and clinical symptoms of neurodegenerative diseases through multiple molecular signaling pathways.

**Table 1:** The role of CAP in neurodegenerative diseases.

AD			
Subject (Animal/Cell/Human)	CAP Processing	Results	Reference
Sprague Dawley (SD) rats (male, 8 months old, 250–350 g)	Dissolved in DMSO (dimethyl sulfoxide) and diluted with 99% phosphate buffer, 1 mg/kg, i.p., for 13 days	Neurodegeneration ↓ Spatial memory impairment ↓ Neuronal degeneration ↓	[21]
3xTg transgenic mice	1 mg/kg, i.p., once for 1 month	Learning memory disorder ↓ Microglia autophagy ↑ Microglia energy metabolism ↑	[46]
TRPV1flox/flox mice; APP/PS1 transgenic mice; Cx3cr1/CreER2 transgenic mice	Feeding standardized food containing 0.01% CAP for 4 weeks	Memory impairment ↓ Impaired microglia metabolism ↓ mTOR signaling ↑ Microglia autophagy ↑ Amyloid	[44]
		pathology ↓ Cellular autophagy ↑	
SD rats (male, 220–280 g)	10 mg/kg, i.g., 1 h before CWS	Spatial memory capacity ↑ Deficits in synaptic plasticity ↓ PP2A activity ↑ Rat hippocampus tau protein hyperphosphorylation ↓	[33]
APP23/PS45 transgenic mice	Daily injections from 1.5 months of age until the end of the behavioral test	Amyloid precursor protein ↓ Hippocampus CA region 1 LTP ↑ AMPAR endocytosis ↓ Memory loss ↓	[45]
APP/PS1 transgenic C57BL/6 mice	Daily intake of CAP in mice at 30 mg/kg 0.01% CAP Random intake from 3 months of age to 9 months of age plus 0.01% CAP	Cognitive impairment ↓ Area fraction and plaque density of total and dense plaques in neocortex and hippocampus ↓ APP processing ↑ Aβ production ↓ Other AD-type pathologic changes ↓	[35]
ApoE mice (5 months old, ApoE 4 HFD-fed); TRPV1-/- mice	1 mg/kg, i.p., one month	Microglia lipid droplet accumulation ↓ Immune dysfunction ↓ Neuronal metabolic impairment ↓ Lipid droplet accumulation ↓ Memory impairment ↓ Loss of neurons ↓	[38]
SD rats (200–500 g, male)	20 mg/kg; 2 days later with 30 mg/kg	Amyloid APP processing ↑ Membrane binding APP ↑	[61]
Human Autopsy Organization		ApoE4 allele causes dysfunctional lipid metabolism in neurons and microglia	[38]
N2a (N2a/WT) cells	250 μM	Amyloid ↓ Generation of ROS ↑ Nrf2 ↑	[42]
N = 338 participants aged 40 or older recruited from the community	Use of an FFQ to collect information on dietary habits related to chili pepper consumption; cognitive functioning was tested using the Chinese version of the MMSE	AD cognitive function in middle-aged and older adults ↑	[35]
Twenty-nine neurodegenerative disease patients (10 ALS patients, 9 PD patients, 5 MSA patients, 3 PSP patients, 1 spinal cerebellar degeneration patient, and 1 Huntington’s disease patient, with a mean age of 71.5 ± 6.0 years)	Soluble thin tablets containing 1.5 μg of CAP or the same amount of placebo were taken 20 min before testing	Signs of dysphagia associated with neurodegenerative diseases ↓	[60]
PD			
C57BL/6 mice (male, 8–10 weeks old)	0.5 mg/kg, i.m., 30 min before MPTP injection	Neuroprotective effects ↑	[50]
Rats	0.001–2.5 mg/kg, i.p.	Inflammatory mediators ↓ Peroxynitrite generation ↓ Oxidative stress ↓ Degeneration of dopamine neurons ↓ The transition of	[51]
		pro-inflammatory M1 microglia/macrophage populations to an anti-inflammatory M2 state ↑	
Wistar rats (male, 220–250 g)	1 mg/kg, i.p., 7 days	Behavioral deficits ↓ Athletic ability ↑ MDA ↓ CAT ↑ SOD ↑	[49]
C57BL/6 mice (males, 8–10 weeks old)	0.5 mg/kg, i.p., 30 min before MPTP injection.	Neuroprotection ↑ Interaction between CBR and TRPV1	[50]
C57 BL/6 mice (males, 23–25 g, 8–10 weeks old)	Different doses of CAP (0.01, 0.1, 0.5, 1, and 2.5 mg kg^−1^), i.p., 1 day, starting 30 min before MPTP injection	DA neuronal damage ↓ Motor behavior ↑ Microglia activation ↓ Pro-inflammatory mediators ↓ Oxidative stress ↓ Astrocytes ↓	[48]
SD rats (female, 240–270 g)	1 mg/kg, i.p., within MFB MPP^+^ 30 min before and 30 min after injection for 6 days	Microglia activation ↓ O^2−^ generation ↓ Neurotoxicity ↓	[52]
SD rats (female, 240–270 g, 10 weeks old)	1 mg/kg, i.p.; single injection for 7 consecutive days, 9 weeks after MPP^+^ injection	Astrocytes ↑ Endogenous CNTF ↑ Mouse rotational asymmetry ↓	[54]
SD rats (female, 10 weeks old, 240–270 g)	1 mg/kg, i.p., 7 days	Phosphorylated p70 S6 K ↑ Survival of dopamine neurons ↑ Motor behavior ↑	[55]
SH-SY5Y cells		Overlap was found between the following seven genes and related	[34]
		pathways: Olr724, C0X1, Gsta2, Rab5a, Potef, Actgl, and Acadsb Actg 1 ↓ Gsta 2 ↑ Apoptosis ↓	
Transgenic Drosophila lines expressing wild-type human synuclein (h-αS) in neurons	20, 40, 80, and 100 μM for 24 days	GSH content ↑ Lipid peroxidation ↓	[56]

↑: promote, increase and positive change; ↓: inhibit, reduce and negative change. Please refer to the literature for details. Abbreviations: CAP: capsaicin; SD: Sprague Dawley; DMSO: dimethyl sulfoxide; APP/PS1: amyloid precursor protein and the human progeria gene 1; mTOR: mammalian target of rapamycin; PP2A: protein phosphatase 2A; APP23/PS45: amyloid precursor protein 23 and the human progeria gene 45; LTP: long-term potentiation; AMPAR: AMPA-selective glutamate receptor; TRPV1: transient receptor potential vanilloid 1; ApoE: apolipoprotein E; FFQ: food frequency questionnaire; MMSE: Modified Mental State Examination; ALS: amyotrophic lateral sclerosis; MSA: multiple system atrophy; PSP: progressive supranuclear palsy; MDA: malondialdehyde; CAT: catalase; SOD: superoxide dismutase; CBR: cannabinoid receptor; CNTF: ciliary neurotrophic factor; GSH: glutathione.

## 3. Cannabinoids

*Cannabis sativa* is a species of the Cannabaceae family native to Asia and is an annual dioecious plant (in rare cases, it can develop hermaphroditic flowers). All species are rich in different biologically active chemical constituents organized into 18 chemical classes, including cannabinoids, alkaloids, terpenoids, and flavonoids produced by secondary metabolism. Their plant extracts were listed in the British and American Pharmacopoeias in the 19th century for their sedative and anticonvulsant effects. Phytocannabinoids are chemically and biologically diverse, showing general antioxidant and anti-inflammatory effects, as well as neuroprotective effects that can be directly mediated through several different biochemical pathways, enabling them to be developed as new therapeutic approaches for neurodegenerative diseases [62]. Cannabis sativa’s biological effects are mediated by two members of the G-protein-coupled receptor family: cannabinoid receptor 1 (CB1R) and cannabinoid receptor 2 (CB2R). CB1R is the predominant isoform in the central nervous system (CNS), expressed in the brain, skeletal muscle, liver, and pancreatic islet cells. It is particularly abundant in brain regions such as the hippocampus, cortex, basal ganglia, and cerebellum, and is expressed in excitatory (dopaminergic) and inhibitory (GABAergic) presynaptic terminals. Many studies have shown that CB1R is neuroprotective against excitotoxicity induced by a variety of stimuli. CB2R is expressed in the testes, spleen and, to a lesser extent, brain [63,64] (Figure 1).

CB1R binding inhibits excitotoxic neurotransmission by blunting presynaptic glutamate release, which has been proposed as the main mechanism underlying CB1R-mediated neuroprotection. It was shown that CB1R protects cultured corpus striatum cells from excitotoxicity via PI3K/AKT/mTORC1/BDNF. Brain-derived neurotrophic factor (BDNF) expression was induced by the selective activation of the BDNF gene promoter IV, an effect mediated by a variety of transcription factors [65].

The study of cannabinoids encompasses three areas of research: endogenous cannabinoids (ECBs), phytocannabinoids, and endogenous cannabinoid-like compounds [66]. The best-studied endogenous cannabinoids are N-arachidonic acid ethanolamine (AEA) and 2-arachidonic acid glycerol (2-AG), both of which are derivatives of arachidonic acid. AEA can be hydrolyzed by fatty acid amide hydrolase (FAAH) and is a full agonist of TRPV1, which is involved in endogenous cannabinoid signaling, while 2-AG is mainly hydrolyzed by monoacylglycerol lipase (MAGL) [63]. Among the nearly 60 different phytocannabinoids identified, cannabidiol (CBD) and Δ^9^-tetrahydrocannabinoid (Δ^9^-THC) have been intensively investigated for their neuroprotective effects in models of AD, PD, and HD [67,68]. Δ^9^-Tetrahydrocannabinolic acid (Δ^9^-THCA) may ameliorate motor deficits and prevent corpus striatum degeneration through the modulation of the PPARγ pathway, resulting in neuroprotection [24].

In studies of AD and cannabinoids, CB1R activation has been shown to prevent amyloid-beta-induced neurotoxicity in several cellular models, although changes in CB1R expression in AD patients or animal models remain controversial [69]. Furthermore, CB1R activation has been reported to be beneficial in animal models of AD with memory deficits and cognitive impairment [63]. In a study using a rat model of AD in which amyloid β (Aβ) injection induced neurotoxicity, CB1R activation was observed to induce a neuroprotective effect on hippocampal CA1 pyramidal neurons via the inhibition of voltage-gated Ca^2^⁺ channels and Ca^2^⁺-activated K⁺ channels [67]. Furthermore, it has been shown that CB2R expression is significantly increased in regions containing microglia associated with neuroinflammatory plaques in the brains of AD patients at necropsy, such as the internal olfactory cortex and parahippocampus [70]. This increase in CB2R expression is thought to be an attempt to counteract the chronic inflammation found in AD, whereas the levels of AEA and its precursors are significantly reduced in the middle frontal and temporal cortex [71]. It has also been shown that glial CB1R is significantly altered in AD mice, with CB1R expression being increased in reactive microglia but remaining constant in astrocytes [72]. In endogenous cannabinoid-related studies, 2-AG has been reported to activate PPAR-γ (peroxisome proliferator-activated receptor-γ) and induce CB1R-dependent anti-inflammatory and neuroprotective effects in response to pro-inflammatory impairments in the neuropathology of Alzheimer’s disease [73] (Figure 2). According to studies on phytocannabinoid extracts, the use of CBD in preclinical models of AD may exert beneficial effects through modulating oxidative and nitrative stress [66]. Another study used cannabidiol to treat a mouse model of AD for 7 days and observed a significant reduction in Aβ expression [67]. Data from a preclinical AD and neurodegenerative disease drug-screening platform suggest that non-psychoactive cannabinoids are potentially leading drug candidates for AD and other neurodegenerative diseases [74].

In studies on PD and cannabinoids, altered expression of CB1R and other components of the endogenous cannabinoid system has been observed, but the exact mechanisms are not yet clear. There is some controversy regarding the expression and role of CB1R. Some studies have shown that CB1R expression is reduced in the basal ganglia of PD patients, that the upregulation of CB1R activity may contribute to the pathological process and worsen the disease, and that the use of CB1R antagonists alleviates motor symptoms in PD models [63,75]. The above findings are not consistent with the conclusion that cannabinoids have neuroprotective effects, but more studies have shown that CB1R’s expression and actions have positive effects in the treatment of PD. CB1R and CB1R–G-protein coupling are increased in the basal ganglia of patients with PD, leading to an increase in CB1R-mediated cannabinoid signaling [75,76]. CB1R and GPR55 receptors are expressed in the corpus striatum and are potential targets for the treatment of PD. The co-expression of CB1R and GPR55 led to significant neuroprotection against PD-inducing MPP^+^ toxins [77]. Δ^9^-Tetrahydrocannabinoid (Δ^9^-THCV), a CB1R antagonist, resulted in an attenuated antimotor effect at doses lower than 5 mg/kg, and studies have shown that the CB1R-selective antagonist SR 141716 (rimonabant) or the blockade of the PI3K/AKT/mTORC1/BDNF pathway can prevent the protective effects of cannabinoids [65,78] (Figure 2). In studies on endogenous cannabinoids, the endogenous cannabinoid 2-AG showed neuroprotective effects against PD-inducing neurotoxins, similar to the effects of cannabinoids [67]. In studies on phytocannabinoid extracts, direct neuroprotective effects were observed in PD-induced SH-SY5Y cell cultures treated with THC (tetrahydrocannabinol) [67]. In a study that used a marmoset model of PD treated with THC and observed a return of motor activity to near pre-disease levels, the authors hypothesized that this positive effect was due to elevated CB1R expression [67]. Some clinical studies have shown that the tetrahydrocannabinol analog nabilone attenuates non-motor symptoms of PD [79]. However, THC medications have mild adverse effects on cognition after acute/short-term use in PD [80]. Notably, cannabinoids have a high potential for abuse and are prone to dependence. Therefore, drugs that modulate ECB (endogenous cannabinoid) levels by blocking their metabolism, such as FAAH and MAGL inhibitors, are therapeutically attractive candidates [63,76,81].

Early studies on HD and cannabinoids reported, for the first time, reduced CB1R expression in the substantia nigra of HD patients based on radiographic autoradiography. Further studies showed that progressive loss of CB1R is an early sign of HD, occurring before the actual neurodegenerative episode and accelerating the progression of HD. This observation was confirmed at the mRNA level as well as by CB1R-associated immunoreactivity in several transgenic mouse models of HD [63]. Further studies in HD cell models showed that CB1R activation protects corpus striatum cells from excitotoxicity by increasing BDNF (brain-derived neurotrophic factor) expression via the PI3K/AKT pathway [63]. Cannabinoids that selectively activate CB1R were neuroprotective in R6/2 mice (a classical genetic model of HD) and quinolinic acid-injured mice (a model driven by excitotoxic events). Cannabinoids that selectively activate CB2R produced the same neuroprotective effect in both models, and they were also active in rats lesioned with malonate, a model with strong glial reactivity and activation of the apoptotic machinery [82]. The loss of CB1R is the primary pathogenic event in HD, and pharmacological strategies that promote CB1R and CB2R signaling may lead to therapeutic benefits, the extent of which likely depends on the extent of the disease [83].

## 4. Piperine

Piperine, a major alkaloid, is widely distributed in nature and can be isolated from the dried fruit extract of pepper; it exhibits pleiotropic effects such as antioxidant, anticancer, anti-inflammatory, antihypertensive, hepatoprotective, neuroprotective, bioavailability-enhancing, and fertility-related activities. In addition, piperine can modulate gastrointestinal disorders, drug-metabolizing enzymes, and the bioavailability of several drugs. Studies have shown that the natural product BioPerine^®^ has thermonutrient activity and bioavailability-enhancing properties. Plant-based compounds, such as curcumin extract, have shown improved bioavailability when administered together with BioPerine^®^ [84]. Piperine has been shown to be useful in the treatment of PD, Alzheimer’s disease, and inflammatory disorders [85].

Piperine is a yellow crystalline solid compound that is biosynthesized from L-lysine and a precursor of cinnamoyl-CoA, a secondary metabolite. After a series of reactions, bioactive piperine is produced from piperoyl-CoA. Piperine is a potential bioavailability enhancer that can be administered with other drugs or herbs, such as curcumin, resveratrol, quercetin, and carbamazepine, to enhance their therapeutic efficacy in neurodegenerative diseases [86]. Piperine is highly lipophilic and has low water solubility, and some studies have demonstrated poor pharmacokinetic properties, such as absorption, bioavailability, and blood–brain barrier permeability; however, recent advances in pharmaceutical technology have overcome several of these limitations, including the issues of bioavailability and blood–brain barrier permeability, improving the compound’s therapeutic efficacy for diseases [86,87].

Piperine has a neuroprotective effect in AD, and some studies have shown that microemulsions can be used as piperine nanocarriers for the treatment of AD and enhance the delivery of piperine to the brain. Piperine alleviates AD symptoms through its antioxidant and anti-acetylcholinesterase activities. In addition, piperine inhibits neuronal inflammation and apoptosis [87]. A study showed that piperine modulated lipid peroxidation as well as enzymatic and non-enzymatic antioxidant levels in pyramidal cells of the hippocampus in an animal model of AD [88].

Piperine can protect the CNS and inhibit neurodegeneration by reducing inflammatory responses, enhancing antioxidant stress responses, modulating autophagy, and blocking amyloid deposition [89]. The molecular basis for the pleiotropic activity of piperine is its ability to modulate multiple signaling molecules and multiple signaling pathways [85]. A study that used a rotarod and rod tests to assess the motor performance of mice with rotenone-induced PD showed that piperine treatment attenuated the rotenone-induced motor deficits and rescued nigrostriatal DA loss by inhibiting the activation of autophagy through the protein phosphatase 2A (PP2A)-mediated inhibition of AKT–mTORC1 signaling, which, in turn, protected SK-N-SH cells and primary neurons from mitochondrial damage induced by rotenone treatment [23]. In addition, piperine exerted neuroprotective effects through antioxidant, anti-apoptotic, and anti-inflammatory mechanisms in 1-methyl-4-phenyl-1,2,3,6-tetrahydropyridine hydrochloride (MPTP)- or 6-hydroxydopamine (6-OHDA)-induced animal models of PD [89,90,91]. In a mouse model of MPTP-induced Parkinson’s disease confirmed through histological examination, piperine treatment attenuated the MPTP-induced deficits in motor coordination and cognitive function, and it also prevented MPTP-induced reductions in the number of nigrostriatal tyrosine hydroxylase-positive cells, while reducing the number of activated microglial cells, expression of the cytokine IL-1β, and oxidative stress [90]. In a 6-OHDA-induced rat model of Parkinson’s disease, piperine treatment significantly inhibited the activation of poly(ADP-ribose) polymerase. Piperine reduced the inflammatory markers TNF-α and IL-1β in rats with 6-OHDA-induced PD, suggesting that piperine exerts a protective effect against 6-OHDA-induced PD through anti-apoptotic and anti-inflammatory mechanisms [91]. A recent experimental study showed that, among 41 derivatives of piperine, 3b exhibited the best neuroprotective effect. The compound 3b exerted cytoprotective effects by inhibiting ROS accumulation and restoring the mitochondrial membrane potential in PC12 cells; the mechanism may be related to the activation of Nrf2 and the expression of corresponding antioxidant proteins, through promoting the entry of Nrf2 into the nucleus, thereby activating cellular oxidative stress defenses and protecting the PC12 cells [89]. Tandem mass tag proteomics analysis showed that piperine had a neuroprotective effect in an SNCA/α-synuclein-induced PD model, by promoting autophagy through P2RX4 activation and attenuating olfactory deficits and delayed locomotor deficits in Thy1-SNCA transgenic mice [92] (Table 2).

Piperine ameliorated experimental autoimmune encephalomyelitis (EAE) in Lewis rats through its neuroprotective, anti-inflammatory, and antioxidant effects, suggesting that piperine has a beneficial effect on the progression of EAE and can be considered as a potential therapeutic target for the treatment of multiple sclerosis (MS). Gene expression analysis of spinal cord tissues showed that piperine treatment reduced the levels of pro-inflammatory cytokines (TNF-α and IL-1β) and iNOS, and it enhanced the expression of IL-10, Nrf2, HO-1 (heme oxygenase 1), and MBP (myelin basic protein, a diagnostic indicator of myelin loss). Piperine supplementation also increased the total antioxidant capacity according to the ferric reducing antioxidant power (FRAP) and decreased the levels of markers of oxidative stress in the central nervous system of rats with EAE. Finally, piperine was found to have anti-apoptotic and neuroprotective effects in EAE by reducing caspase-3 (an apoptotic marker) and enhancing BDNF and NeuN (neuron-specific nuclear protein) expression [93]. Piperine, as a potent inhibitor of dihydroorotic acid dehydrogenase (DHODH), demonstrated strong prophylactic and therapeutic effects in MOG (myelin oligodendrocyte glycoprotein)-induced experimental autoimmune encephalomyelitis (EAE) by restricting the infiltration of inflammatory cells into the CNS and preventing myelin and blood–brain barrier (BBB) disruption, proving a novel role for piperine in the treatment of MS [94]. All of the above studies suggest that piperine has neuroprotective effects, suggesting that piperine could be an effective drug for the treatment of neurodegenerative diseases (Table 2).

Furthermore, it has also been shown that a combination of quercetin and piperine exerted neuroprotective effects in a rat model of PD induced by rotenone and iron supplementation through antioxidant and anti-inflammatory mechanisms [95]. Combination therapy with quercetin and piperine also significantly ameliorated MPTP-induced behavioral abnormalities in rats, reversed abnormal neurotransmitter alterations in the corpus striatum, and attenuated oxidative stress and inflammatory responses in the corpus striatum [90]. Piperine has been reported to enhance the bioavailability of various compounds, including quercetin, by inhibiting glucuronosyltransferases, P-glycoprotein, and drug-metabolizing enzymes [95]. Studies have shown that curcumin, in combination with piperine, can be neuroprotective in a 3-NP (3-nitropropionic acid)-induced HD model, and that piperine significantly enhances the protective effect of curcumin [96]. The above studies suggest that piperine can also be used as a bioavailability enhancer in combination with quercetin or curcumin, exhibiting strong neuroprotective effects (Table 2).

Piperine and its derivatives play a neuroprotective role through antioxidant, anti-inflammatory, autophagy-regulating, and other mechanisms. However, the mechanisms of piperine’s action have not been fully characterized, and most of the studies are still at the level of pharmacological effects, signaling molecules, and specific signaling pathways (e.g., the AKT–mTORC1 signaling pathway); they cannot explain the connections between relevant signaling molecules, signaling pathways, and specific drug targets in the context of the overall explanation of its pharmacological effects. The connection between signaling molecules, signaling pathways, and specific drug targets cannot yet be explained from a holistic point of view; therefore, further studies are needed to clarify piperine’s mechanism of action. In addition, further exploration of its derivatives to improve the neuroprotective activity and selectivity of piperine will be of great significance for the treatment of neurodegenerative diseases.

**Table 2:** The roles of piperine in neurodegenerative diseases.

AD			
Subject (of an Experiment) (Animal/Cell)	Piperine Processing	Results	Reference
Wistar rats (male, 3 months old, 250 ± 50 g)	10 mL/kg, i.g., for 21 consecutive days	Memory performance in rats ↑ Improvement in brain function ↑	[88]
PD			
Thy1-SNCA	25, 50, and 100 mg/kg	Olfactory deficits ↓	[92]
Transgenic mice; SK-N-SH cells	p.o., for 6 weeks; 0.2, 1, 5, 25, and 125 μM beginning 24 h	Motor deficits ↓ Cell viability in SK-N-SH cells ↑ Degradation of human SNCA ↑ Autophagosomal–lysosomal membrane fusion ↑ Autophagic flux ↑	
H_2_O_2_-induced PC12 cells	12.5, 25, and 50 mM for 24 h	Clear ROS ↑ Activation of Nrf2 ↑ Related phase II antioxidant enzymes	[89]
		HO-1 and NQO1 ↑	
C57BL/6 mice (male, 3 months old)	25 mg/kg, 50 mg/kg, p.o., for 4 weeks	Motion defects ↓ Loss of black matter DA ↓	[23]
Wistar rats (male,	2.5 mg/kg, p.o., alone,	Antioxidant, anti-	[90]
250–280 g)	with piperine 2.5 mg/kg and	inflammatory, and neuroprotective effects ↑	
C57 BL/6 mouse model (male, 18–20 g)	10 mg/kg, p.o., for 15 days, including 8 days of pretreatment.	Deficits in motor coordination and cognitive function ↓ Decrease in the number of nigral tyrosine hydroxylase-positive cells ↓ Number of activated microglia ↓ Expression of cytokine IL-1β and oxidative stress ↓	[95]
Wistar rats (male	10 mg/kg bwt for 15 days	Lipid peroxidation ↓	[91]
adults, 250–300 g)	before injury in rats, p.o.	Cytochrome C release ↓ Activation of cysteinyl	
		asparaginase-3 and cysteinyl asparaginase-9 ↓ Activation of poly(ADP-ribose) polymerase ↓	
HD			
Wistar rats (male, 250–280 g)	Piperine at 25 and 50 mg/kg, p.o.; CUR at 25 mg/kg, p.o., with piperine at 2.5 mg/kg, p.o., once a day for 21 days	Motor deficits in rats ↓ Neuroprotective effects of combined applications ↑ Functional recovery in behavioral, biochemical, neuroinflammatory, and neurochemical alterations in rats ↑	[96]
EAE			
Lewis rats (female adults, 180–200 g)	5 mg/kg/day, i.p., from days 8 to 29	Neurological deficits ↓ EAE disease progression ↓ Activation of microglia and astrocytes ↓	[93]
		Immune cell infiltration ↓	
Jurkat T cells	0, 0.025, 0.05, 0.1, 0.25, 0.5, 1.0, 2.5, 5, 10, 25, and 50 μM	Preventive and therapeutic role of performance in EAE ↑ T-cell proliferation in a	[94]
		DHODH-dependent manner ↓	

Abbreviations: PIP: piperine; SK-N-SH cells: human neuroblastoma cells; SNCA: recombinant synuclein alpha; RS: reactive oxygen species; Nrf2: nuclear factor erythroid 2-related factor 2; NQO1: recombinant NADH dehydrogenase, quinone 1; HO-1: heme oxygenase-1; DA: dopamine; EAE: experimental autoimmune encephalomyelitis; DHODH: dihydroorotate dehydrogenase.

## 5. Curcumin

Curcumin (CUR) is the main polyphenolic compound in turmeric rhizomes, with the chemical formula C_21_H_20_O_6_, and its main metabolite is 1,7-bis(4-hydroxy-3-methoxyphenyl) heptane-3,5-dione, tetrahydrocurcumin (THC) [97,98]. CUR exists mainly as an enol in alkaline media and as a ketone in acidic media, with keto–enol interconversion isomerism [99,100]. CUR is a hydrophobic molecule, insoluble in polar and neutral solvents and soluble in organic or hydrophobic solvents [78]. Due to its hydrophobic tendency, CUR has poor pharmacokinetics and is readily degraded by the liver [101]. As plant-derived components with antioxidant and anti-inflammatory properties, CUR and its metabolites and derivatives have been shown to have preventive and therapeutic effects in a variety of diseases, such as tumors, post-ischemic brain injury, cardiovascular and cerebrovascular diseases, diabetes mellitus, and neurological inflammation [101,102].

CUR achieves its protective effect on neurodegeneration mainly through the Wnt/β-catenin pathway and phosphatidylinositol-3 kinase/protein kinase B signaling pathway (PI3K/AKT signaling pathway). The Wnt protein is a paracrine protein that can bind to frizzled (Frz) and induce a series of intracellular signaling pathways, the most important of which is the classical conduction pathway of the Wnt protein, i.e., the Wnt/β-catenin pathway, which is important for the homeostasis and regeneration of neural stem cells. The classic Wnt protein transduction pathway, i.e., the Wnt/β-catenin pathway, is also extremely important for neural stem cell homeostasis and regeneration [103]. The Wnt/β-catenin pathway consists of three main steps: Wnt signaling, β-cyclin’s stabilization and accumulation in the cytoplasm, and the activation of Wnt target genes in the nucleus [104,105]. When Wnt signaling is not activated, β-cyclins are degraded by a protein destruction complex containing glycogen synthase kinase-3 (GSK-3) [103]. Another important signaling pathway is the PI3K/AKT/mTOR signaling pathway, which is closely associated with nerve regeneration and anti-apoptotic activity, and whose dysfunction induces neuronal death [106]. The mammalian target of rapamycin (mTOR) is an atypical serine/threonine protein kinase that plays an important role in inhibiting apoptosis and promoting cell proliferation [22]. In addition, BDNF synergistically maintains neuronal cell activity by activating PI3K/AKT to inhibit apoptosis through several pathways [106,107]. Meanwhile, it has been shown that AKT can phosphorylate Ser 21 of GSK-3α or Ser 9 of GSK-3β, inactivating them, thereby reducing GSK-3’s inhibition of β-cyclin and nuclear factor erythroid-2-related factor 2 (Nrf2), resulting in certain neuroprotective effects [108,109,110].

Based on the above signaling pathways, CUR is neuroprotective against AD. The current evidence shows that curcumin inhibits Aβ production, partially through the downregulation of the PI3K/AKT/mTOR signaling pathway [22,111]. CUR also protects neuronal cells by regulating the expression of inflammatory factors (such as IL-10) and macrophage differentiation and polarization [112]. It also attenuates neuroinflammation in AD by inhibiting the HMGB1–RAGE/TLR4–NF-κB signaling pathway, activating PPRA-γ [113]. Another study showed that CUR significantly reduced co-stimulatory molecules and polarized/repolarized macrophages toward the M2 phenotype; CUR-treated macrophages were more efficient in the mannose receptor antigen capture pathway and endocytosis [114]. In addition, CUR protects mitochondria by preventing ΔΨm loss, inhibiting cysteine asparaginase-3 (caspase-3) activation, and altering Bcl-2 family expression, as well as decreasing intracellular ROS production, thus protecting neuronal cells from cytotoxicity [101,115]. The above studies demonstrate that CUR protects mitochondria through an extremely broad spectrum of activities. These studies also demonstrate that CUR mediates neuroprotection in AD through a wide range of pathways; however, the chemical properties of CUR have made it difficult to conduct clinical trials [116]. In order to further enhance the therapeutic effect of curcumin, researchers have combined curcumin with different carriers to make different complexes containing the active components of CUR, in order to improve the low oral bioavailability of CUR and to increase the efficiency by which its active components act on targets. Examples of such complexes include exo-columnar liposomes composed of CUR, chitosan–polylactic acid–hydroxyacetic acid copolymer nanoparticles (CUR-CS-PLGA-NPs), and CUR encapsulated in hydroxypropyl-β-cyclodextrin (CUR/HP-β-CD encapsulation). The above CUR-containing composites exhibited excellent pharmacokinetics, with a significant enhancement of the drug utilization rate. Compared with CUR, there was a significant increase in drug utilization, while related studies have demonstrated that these complexes have excellent performance in reducing the aggregation and accumulation of Aβ in relevant brain regions, alleviating neuroinflammation, protecting neuronal cells, inducing neuronal differentiation gene expression, and ameliorating behavioral deficits, cognitive deterioration, and memory deficits in AD animals [115,117,118,119,120,121,122]. CUR has been shown to be a neuroprotective agent. In addition to the neuroprotective effects of CUR itself, the metabolites and derivatives of CUR are also effective in AD, and they cross the blood–brain barrier more efficiently, showing superior protective and therapeutic effects. Tetrahydrocurcumin, the main metabolite of CUR, can inhibit microglial apoptosis through the Ras/ERK signaling pathway, increase the phagocytosis and internalization of Aβ, and reduce the Aβ load in the hippocampus. In addition, it can rescue the decline in neuronal cell viability and protect learning and memory in APP/PS1 mice [98]. The polyphenolic curcumin derivatives GT 863/PE 859 inhibit the cleavage of γ-secretase in a substrate-dependent manner, thereby inhibiting protein N-glycosylation and reducing Aβ production [123].

**Figure 3:**
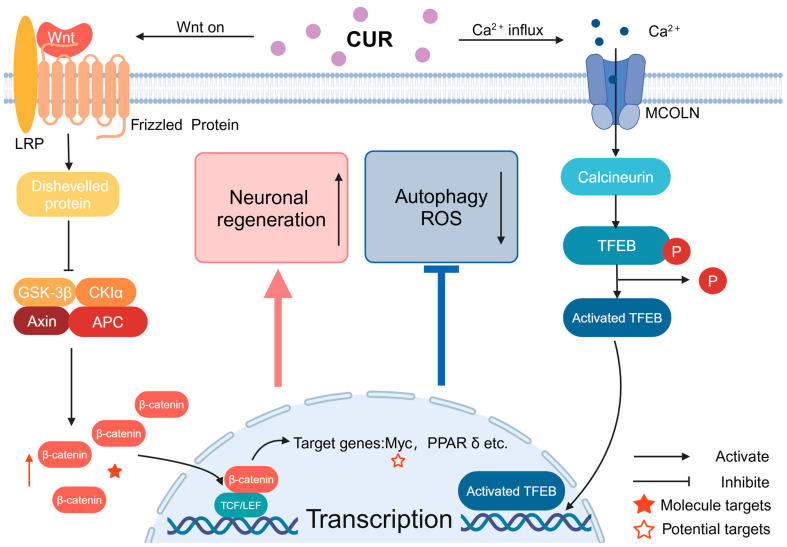
The signaling of Wnt and calcium ions through MCOLN: CUR can activate Wnt signaling way by increasing the stability of β-cyclin, leading to its accumulation in the cytoplasm. Following this, the Wnt signaling pathway triggers the activation of Wnt target genes within the nucleus, which ultimately contributes to neuroprotection. CUR also can facilitate the entry of calcium ions into the cell through MCOLN, activate TFEB, and the activated TFEB enters the nucleus, promoting the transcription of related genes.6. Nicotine.

The neuroprotective effects of CUR in PD are specifically characterized by the maintenance of mitochondrial homeostasis, the inhibition of astrocyte activation and inflammatory responses, and a reduction in α-syn synthesis and aggregation [124]. CUR improves mitochondrial respiratory chain function and reduces mitochondrial ROS production in PD mice by activating the Wnt/β-catenin pathway and the PI3 K/AKT signaling pathway, thereby inhibiting oxidative stress and maintaining neuronal activity [97,104,122,123]. In addition to the protective mechanisms described above, CUR and its derivatives have been shown to maintain DA neuronal activity in the substantia nigra of mice by inhibiting the mucolipid 1/calcium/calmodulated neural phosphatase/transcription factor EB (MCOLN1/calcineurin/TFEB) pathway, increasing the expression of TFEB, and attenuating the cytotoxicity of 6-OHDA [125] (Figure 3). CUR and cyclohexylcyclohexane CNB-001, a novel pyrazole derivative of CUR and cyclohexylbisphenol A, significantly upregulated the expression of the anti-apoptotic protein Bcl-2 and attenuated the expression of pro-apoptotic proteins, suggesting that CNB-001 may provide neuroprotection by modulating the function of the Bcl-2 family [126]. In another study, the CUR agonism of human α7-nicotine-acetylcholine receptor (α7-nAChR) decreased the mortality of DA neurons in the substantia nigra and significantly reduced 6-OHDA-induced dyskinesia in mice with PD, suggesting that the activation of α7-nAChR by CUR has neuroprotective effects in PD [127]. Meanwhile, in a study of a Drosophila model of PD, treatment with curcumin significantly improved locomotor performance and neurodegeneration [128]. CUR may also exert some protective effects against PD by modulating the gut microbiome–metabolite axis [129]. Researchers have made many efforts to enhance the utilization of CUR, such as making rabies virus glycoprotein (RVG) peptide-modified exosomes/curcumin/phenylboronic acid-poly(2-dimethylamino)ethylacrylate nanoparticles/small interfering RNA-targeted SNCA through engineered core–shell hybridization (REXO-C/ANP/S), utilizing trivalent ferric ions and CUR ligands to make ultra-small nanoscale coordination polymers (NCPs), and preparing novel CUR oil solutions and acid nanocarriers alone or in combination with desferrioxamine to enhance the bioavailability of CUR, and the above complexes have been shown to protect DAergic neurons, alleviate neuroinflammation, and mitigate dyskinesia and memory deficits in both in vivo and in vitro models of PD [130,131,132,133] (Table 3).

In conclusion, the mechanism by which CUR protects against neurodegeneration has been thoroughly studied, and researchers are working on the development of more efficient CUR delivery regimens or CUR analogs, which will have excellent application prospects.

Nicotine, which has a strong flavor, is a potent parasympathetic alkaloid found at high levels in the tobacco plant and extracted from its leaves that acts as an nAChR agonist, inhibiting amyloid β (Aβ) formation and the neurotoxic effects of excitatory glutamate, and enhancing nerve growth factor effects through the stimulation of the nicotinic receptor α4β2 [145,146]. The subunits of mammalian neuronal nAChR range from α2-α7, α9, and α10 to β2-β4, and they are formed by various combinations of homodimeric and heterodimeric receptor subtypes with different functions [147].

**Figure 4:**
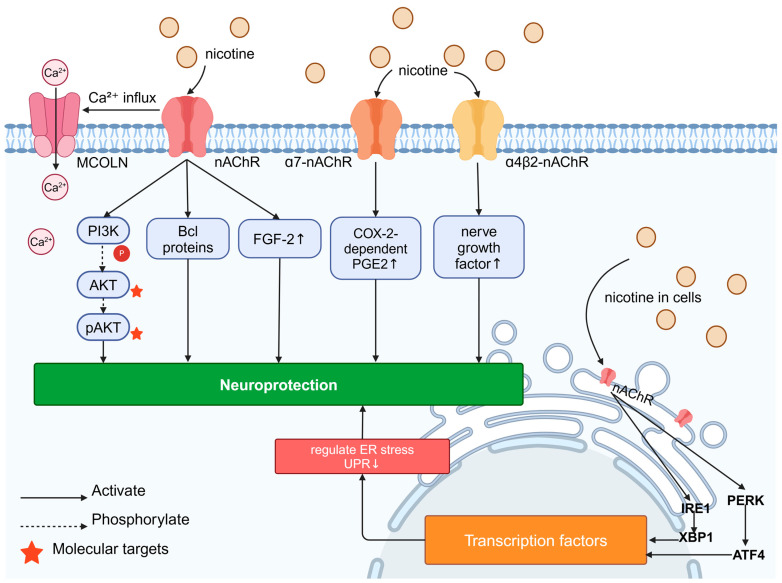
Signaling pathways for neuroprotective effects of nicotine: Nicotine’s “outside–in” neuroprotective mechanism is its activation of surface nAChR, leading to Ca^2^⁺ influx and the upregulation of FGF-2 expression; nAChR activation also prevents neurodegeneration through mechanisms involving the activation of pro-survival signaling factors such as the PI3K, AKT, and Bcl proteins in the brain. Nicotine can increase the enhancement of nerve growth factor induced by α4β2-nAChR; in astrocytes cultured in vitro, the activation of α7-nAChR leads to the COX-2-dependent production of PGE2, thereby exerting neuroprotective effects. Nicotine’s “inside–out” neuroprotective mechanism involves its activity as a molecular chaperone of nAChR in cells, which can modulate protein kinase R-like ER-localized eukaryotic initiation factor 2α kinase (PERK)-activating transcription factor 4 (ATF4) and inositol-requiring enzyme 1 (IRE1)-X-box binding protein 1 (XBP1), thereby altering ER stress/the unfolded protein response (UPR), leading to transcriptional changes and neuroprotection.

The neuroprotective effects of nicotine have been well documented. In astrocytes cultured in vitro, nicotine treatment inhibited cytokines (e.g., IL-6, TNF-α, and IL-1β), downregulated key inflammatory mediators (e.g., IL-8 and BuChE), and exerted neuroprotection through the activation of α7-nAChR, leading to COX-2-dependent PGE2 production, which delayed neurodegeneration [148]. Some studies have hypothesized an “outside–in” mechanism of neuroprotection, in which nicotine activates the surface nAChR, leading to Ca^2+^ influx and transcriptional changes, such as the upregulation of FGF-2 mRNA expression (Figure 4). The *FGF-2* gene is involved in the nAChR mechanism mediating neuronal survival and nutrition, and nicotine may also mediate neuronal survival through α7-nAChR activation. Nicotine also exerts neuroprotective effects through an “inside–out” pharmacology. Nicotine-induced nAChR chaperoning can alter the endoplasmic reticulum (ER) physiology and the ER stress/unfolded protein response (UPR). Intracellular interactions between nicotine and nAChRs are sufficient to modulate ER stress and reduce the UPR, and these effects are associated with an increase in fibroblast growth factor 2 (FGF-2) mRNA expression. It has been shown that these effects are associated with an increase in the number of Sec24d molecules at the endoplasmic reticulum exit site, the translocation of ATF6 (activating transcription factor 6), and the phosphorylation of eIF2β (protein kinase R-like ER-localized eukaryotic initiation factor 2 β), and it has been proposed that nicotine’s concomitant effects on nAChRs enhance the export of a4b2 nAChR from the ER, which leads to a generalized increase in ER exit sites and, ultimately, to the upregulation of the plasma membrane. In addition, the authors hypothesized that this process reduces the requirement for a general protease-inhibitory mechanism in the ER, thereby altering ER stress (an often-cited mechanism of toxicity in PD) and, ultimately, exerting neuroprotective effects [28,149,150]. It has been shown that nicotine inhibits H_2_O_2_-induced fine damage to immortalized hippocampus HT-22 cells through the activation of the α7-nAChR/Erk 1/2 signaling pathway, exerting neuroprotection through antioxidant effects [25]. The activation of nAChR has been shown to prevent neurodegeneration through mechanisms involving the activation of pro-survival signaling factors in the brain, such as the PI3K, AKT, and Bcl proteins [151].

Nicotine may exert neuroprotective effects in AD. Studies have shown that nicotine activates the nAChR, which helps to protect against Aβ-induced toxicity, while scavenging the peptide—an effect that contributes to the treatment of AD, with a potential role for the nAChR as a therapeutic target [152]. Nicotine, as an nAChR agonist, confers cognitive benefits through a variety of mechanisms, including the stimulation of cholinergic pathways, modulation of inflammation, and buffering of amyloid [153]. Another study found that the nicotine treatment of a mouse model of AD (APPsw transgenic mice carrying the Swedish mutation in the human amyloid precursor protein) effectively reduced amyloid β-peptide aggregation in the brain, with a significant reduction of more than 80% in positive plaques in the brain [154]. Studies have shown that long-term nicotine treatment prevents cognitive impairment in young and old APP/PS1 transgenic mice. Nicotine-stabilized β-linker proteins prevented early postsynaptic and late presynaptic damage as well as the Aβ-induced loss of β-linker proteins through a mechanism dependent on α7-nAChR activation. In addition, the activation of classical Wnt/β-collagen signaling upregulates α7-nAChR expression [26]. Furthermore, nicotine enhances nerve growth factor action by stimulating the nicotinic receptor α4β2 [145]. However, many compounds in tobacco smoke are toxic and oxidative stress inducers, rendering nicotine unable to exert neuroprotective effects. Free radicals are thought to play a key role in promoting the development of AD and cognitive dysfunction in smokers, and oxidative stress regulates APP processing by altering β- and γ-secretase activity and promotes Aβ production through the JNK and PKR–eIF2α signaling pathways; cigarette smoking-associated cerebral oxidative stress is a potential mechanism that promotes AD pathology and increases the risk of AD [155,156].

Nicotine may play a neuroprotective role in PD. A study showed that smoking reduced the risk of developing PD by 74%, with a negative correlation between the two [157,158]. The above data suggest that tobacco may have neuroprotective effects in PD and contain a neuroprotective substance. A prospective study showed that women with higher dietary nicotine intake (dietary nicotine intake was calculated based on the intake of chili peppers, tomatoes, processed tomatoes, potatoes, and tea) had a lower risk of PD than those with lower intake, and that one of the mechanisms underlying this was the close anatomical relationship between the cholinergic and dopaminergic neurotransmitter systems in the corpus striatum [9,10,11]. There is a close anatomical relationship between the cholinergic and dopaminergic neurotransmitter systems in the corpus striatum [159]. Another hypothesis is that nicotine exerts its effects by altering the composition of the gut microbiota, thereby reducing gut inflammation. Reduced inflammation would lead to less misfolding of α-syn, thereby reducing the abundance of the misfolded form that might be able to enter the central nervous system and thereby lead to disease and neurodegeneration [151,159,160]. In addition, studies have also found a significant relationship between an increased intake of chili peppers and reduced risk of PD, suggesting that nicotine and other components of chili peppers may have synergistic effects [159]. Preclinical studies have shown that nicotine metabolites are norcholinergic modulators that produce beneficial effects by stimulating cholinergic neural pathways, displaying pro-cognitive and neuroprotective properties associated with the positive regulation of the cholinergic and dopaminergic systems. Nicotine derivatives such as cotinine have great potential to be effective agents for the prevention and alleviation of neurological symptoms seen in subjects with Parkinson’s disease [151]. A study showed that SIRT6 plays a pathogenic and pro-inflammatory role in PD, and that nicotine can provide neuroprotection by accelerating its degradation, suggesting that the inhibition of SIRT6 may be a promising strategy for ameliorating PD and other neurodegenerative diseases [161]. The possible therapeutic effects of nicotine are mediated through the activation of the astrocyte nAChR; astrocyte calcium mobilization decreases pro-inflammatory cytokine release and increases astrocyte glial-derived neurotrophic factor synthesis, suggesting that nAChR expressed on astrocytes may be a better therapeutic target for the treatment of PD [162]. α7-nAChR may serve as a therapeutic target, and nicotine inhibits H_2_O_2_-induced astrocyte apoptosis through the mitochondrial pathway by stimulating α7-nAChR, which also reduces the progression of PD and decreases L-dopa-induced dyskinesia (LID) [163,164]. The endogenous α7-nAChR mechanism plays a key role in mouse models of PD by regulating Wnt/β-collagen signaling [27]. PD mice lacking α5-nAChR demonstrated reduced dyskinesia after nicotine administration [165]. However, a case–control study presented a different view, with data that support the idea that PD patients are more likely to quit smoking than controls. These findings are consistent with a reduced responsiveness to nicotine during the prodromal phase of PD, and they also suggest that the apparent “neuroprotective” effect of smoking observed in epidemiological studies is due to reverse causality [166].

## 6. Other Substances

In addition to the substances mentioned above, researchers have found that other natural plant extracts with pungent odors or tastes have similar potential neuroprotective effects.

Allicin, diallyl trisulfide (DATS), is an organic sulfide extracted from garlic. Allicin can protect the function of mitochondria and the endoplasmic reticulum by reducing lipid peroxidation, and it can inhibit oxidative stress and inflammation by inhibiting the secretion and gene expression of the pro-inflammatory factors TNF-α, IL-1β, and MCP-1, enhancing PERK/Nrf2 signaling, increasing the expression of ApoE and p38 MAPK, upregulating antioxidant enzymes and detoxification enzymes, and scavenging free radicals and oxidative substances, improving learning and memory impairment in rats [167,168,169,170].

Glucosinolates (GL) are alkaloids found in cruciferous plants that emit a pungent, irritating odor and can be hydrolyzed into neuroprotective isothiocyanates (ITCs), such as sulforaphane (SFN), the main source of the pungent odor in raw radish, and sinapic acid (EA), the source of the choking odor of mustard [171]. ITCs can directly interact with sulfhydryl residues on Kelch-like ECH-associated protein 1 (Keap1), blocking Nrf2-mediated inflammatory responses, thereby protecting nerve cells from inflammatory responses [172]. In addition to this, SFN inhibits the formation of Aβ and promotes the degradation and clearance of Aβ, showing potential for the treatment and prevention of AD [173,174,175]. EA is a major component of rapeseed, mustard, and canola oils, and it is widely consumed in Asian countries, mainly through the consumption of cruciferous vegetables, including mustard and rapeseed oils [176,177]. There is extensive liposome remodeling in EA-treated cells, which leads to the incorporation of very-long-chain fatty acids (VLCFAs) and altered γ-secretase function in cell membranes, which, in turn, affects APP processing and increases Aβ_37_ and/or Aβ_38_ secretion, thereby reducing pathological Aβ production and aggregation [178]. Studies have shown that EA can inhibit the in vitro activity of essential enzymes (acetylcholinesterase and tyrosinase) involved in the pathological progression of AD and PD [179]. Erucamide, a bioactive fatty acid amide derivative of EA obtained from radish leaves, similarly exhibited potent acetylcholinesterase-inhibitory activity [180]. In addition to this, EA can be neuroprotective against AD by increasing the expression of PI3K, CREB (cAMP-response element-binding protein), and ERK in neuronal cells, elevating the level of catalase, and decreasing the level of cholinesterase. EA can also be converted into the key component of myelin sheaths, neuronic acid, which is hypothesized to have a possible regenerative effect on myelin [181]. In addition, EA has been shown to be neuroprotective in X-linked adrenoleukodystrophy (X-ALD), a demyelinating lesion of the central nervous system [182,183].

Cumin is slightly bitter and spicy, with a distinctive crisp odor, and one of its main components—p-isopropylphenyl aldehyde (cuminaldehyde), or cumin aldehyde—inhibits α-syn fibrosis [184]. Another study showed that cumin aldehyde has an anti-amyloid effect, which suggests that it may affect the pathological process of neurodegeneration [185].

At the same time, two beverages with distinctive aromas—namely, coffee and tea—have certain active ingredients that show some therapeutic potential for neurodegenerative diseases. Coffee, with its distinctive bitter aroma and full-bodied flavor, is loved by people around the globe and is the most consumed beverage in the world, with its unique flavor derived from caffeine. Tea, on the other hand, is a more traditional and milder beverage, second only to coffee in terms of global average annual consumption, and derives its bitter odor mainly from epigallocatechin-3-gallate (EGCG) [186,187]. A prospective cohort study showed that coffee and caffeine-containing tea consumption had a preventive and palliative effect on at least one neurodegenerative disease [188]. Studies have shown that caffeine intake is negatively correlated with corpus striatum volume, the availability of DAT in the corpus striatum, and the formation of Lewy bodies, while being positively correlated with nicotinamide adenine dinucleotide (NAD) availability in PD patients, and these studies suggest that caffeine has a potential ameliorative effect on pathological changes and clinical symptoms in PD patients [189,190,191,192]. Meanwhile, other studies have shown that the active ingredient of tea, EGCG, can inhibit the aggregation of α-syn, Aβ, and huntingtin, as well as participating in the regulation of neuronal inflammatory processes and the activation of pro-neuronal survival signaling pathways, which can have a certain therapeutic effect in AD, PD, and HD [193,194,195].

In summary, many kinds of natural plant extracts with specific odors have been found to have therapeutic potential for neurodegenerative diseases, but the evidence mainly comes from laboratory studies, and there is still a lack of sufficient preclinical and clinical studies to elaborate the neuroprotective effects of stimulant extracts—a gap that urgently needs to be filled.

## 7. Discussion

In this review, we describe the nature and mechanisms of action of several stimulating flavor components that protect against neurodegeneration, as well as the laboratory and clinical evidence demonstrating their neuroprotective effects.

A comparison revealed that different stimulating flavor components could exert neuroprotective effects by activating the same signaling pathways: CAP, piperine, CUR, and cannabinoids could all induce cellular autophagy by inhibiting PI3K/AKT/mTOR pathway activity. Piperine, CUR, and cannabinoids can protect cells from toxic proteins by upregulating the expression of the downstream signaling molecule BDNF. The PPARγ pathway is also widely involved in the regulation of neuroprotective mechanisms. It is hypothesized that one of the reasons that different stimulating flavor components can exert neuroprotective effects through the same signaling pathway is that certain flavor components have the same metabolites, such as THC, which is extremely efficient in neuroprotection as a metabolite of CUR and piperine. We also found that different stimulating flavor components can act on common receptors to exert neuroprotection: both CUR and nicotine block α7-AChR, and both CAP and cannabinoids block TRVP1, which is involved in neuroprotective cell signaling [32,39,63,127,148]. In addition to the action of different substances on common receptors, nicotine, curcumin, and caffeine have been found to exert neuroprotective effects through intestinal mechanisms, mainly by altering the composition of the gut microbiota [129,151,159,160].

In addition, the combination of different stimulating flavor components showed synergistic neuroprotective effects: the combination of piperine with CUR and quercetin significantly enhanced the neuroprotective effects of both substances [95,96], and there was also a significant relationship between an increased intake of chili peppers and a reduced risk of PD, suggesting that the nicotine in chili peppers may have a synergistic effect with CAP, although the exact mechanism has not yet been clarified [159].

Currently, the neuroprotective effects of stimulant flavor ingredients have been widely and comprehensively studied, but there has not yet been a single preventive or therapeutic drug containing a stimulant flavor ingredient that has actually been applied in clinical use, mainly because the mechanism underlying the disease itself remains unclear, the mechanisms underlying the stimulant flavor ingredient’s protective effects have not yet been agreed upon, and there is a lack of clinical trials. In addition, the abovementioned stimulant flavor components have low bioavailability and high hepatic and intestinal circulation clearance; therefore, the search for more efficient derivatives or complexes and the selection of a convenient and fast route of administration will bring new breakthroughs in the development of drugs to combat neurodegeneration. This will require cross-disciplinary cooperation among pharmacology, clinical medicine, chemical engineering, and other disciplines. To date, the research on enhancing the utilization of CUR has been more comprehensive, and researchers have made many attempts, such as using engineered core–shell hybridization to make REXO-C/ANP/S, preparing novel CUR oil solutions, and binding different ligands to CUR, providing a reference and directions for the preparation of other stimulating flavor component complexes.

## 8. Summary

The incidence of neurodegenerative diseases is increasing year by year worldwide, seriously affecting the quality of life of patients and placing financial burdens on patients’ families. However, current treatments for neurodegenerative diseases are still limited. Some studies have found that pungent flavor compounds have potential protective effects in neurodegenerative diseases, which brings new hope for their treatment. Therefore, this article summarizes the mechanisms of action of several pungent flavor compounds that have potential therapeutic effects in neurodegenerative diseases. We identified the similarities among these pungent flavor compounds to provide a reference for the further exploration of the efficacy of these substances and the development of new drugs for neurodegenerative diseases. Furthermore, the aforementioned pungent flavor components exhibit low bioavailability. Consequently, the pursuit of more potent derivatives or complexes, coupled with the identification of a convenient and rapid administration route, could mark significant advancements in the development of neurodegenerative therapeutics. This collaborative venture necessitates the integration of expertise across multiple disciplines, including pharmacology, clinical medicine, chemical engineering, and others. According to our discussion, we could find ways to explore more efficient drugs that can make patients better. All in all, this review provides reference and direction for the treatment of neurodegenerative diseases.

## Figures and Tables

**Table 3:** The roles of CUR in neurodegenerative diseases.

AD			
Subject	Curcumin	Results	Reference
ICR mice (male, 6 weeks old, 25–35 g)	2.5 mg/kg, p.o. (low dose, LD); 5 mg/kg, p.o. (high dose, HD); 14 days	GSH ↑ CAT ↑ SOD ↑ GPx activity ↑ (in high-dose group) MDA ↓ AChE ↓	[134]
C57BL/6 wild-type mice expressing chimeric mouse/human amyloid precursor protein; APP/PS 1 double-transgenic mice (male)	0.5% wt/vol CMC-Na solution, i.g., APP/PS1 mice, representing the APP/PS1 + V group, or with THC (i.g.) at a daily dose of 400 mg/kg, representing the APP/PS1 + THC group	Gab 2 ↑ K-Ras ↑ Bag 1 ↑ TGF-β1 ↑ Ccnd 2 ↑ Caspase-3 ↓ TNF-α ↓ PARP 1 ↓ Cleaved-PARP1 ↓	[98]
5xFAD mice and wild-type mice (6 months old, 12 months old)	SLCP, p.o., 100 mg/kg, 2 months	Fixed and degenerating cells in prefrontal cortex (PFC), entorhinal cortex (EC), CA 1, CA 3, and inferior torus complex (SC) domains ↓; Dendritic branching and dendritic spine density from primary, secondary, and tertiary apical and basal branches in PFC, EC, CA1, and CA3 ↑	[135]
Swiss albino mice (male, 18–22 g)	S-SNEDDS, o.p., 25 and 50 mg/kg	Learning and memory capacity ↑	[116]
APP/PS1 transgenic mice (male, 3 months)	100 mg/kg/d, o.p., for 5 months starting at 4 months of age	Evasion of latency ↓ HMGB 1 protein expression ↓ Late glycosylation end product-XI-specific receptor (RAGE) ↓	[136]
		Toll-XI-like receptor-XI 4 (TLR 4) ↓ Nuclear factor κ B (NF-κB) ↓ p65↓ Spatial learning memory ↑ Neuroinflammation ↓	
C57 BL/6 mice (male, adult, 20–25 g)	CUR solution, CUR-CSPLGA-NPs, and CUR/HP-β-CD envelope by intranasal route, 6 μL	In vivo pharmacokinetic studies have shown that the intranasal route of administration enhances the distribution of CUR in the brain	[120]
APP/PS1 double transgenic mice (6 months old)	160 ppm or 1000 ppm for 6 months	Memory and cognitive impairment ↓ Aβ42 production ↓ Autophagy ↑ Hippocampus CA Zone 1 Beclin 1 ↑ Hippocampus CA region 1 and cortex LC 3 I/II ↑ p-Akt and p-mTOR ↓	[22]
CHO cells stably expressing human wild-type APP 751; CHO-APP cells	10μΜ, 10μΜ curcumin derivative, or 3 μΜ GT 863	Aβ40 and Aβ42 ↓ C83 and C99 ↑ γ-Cleavage ↓ N-glycosylation of proteins ↓	[137]
Cells stably expressing mouse Notch1αE		Inhibition of the mannose-pruning step in the protein N-glycosylation pathway ↓	
Recombinant Tau expressed in *E. coli* BL21 * (specific derivative strain) cells (hTau40WT)	100 μM curcumin, artemisinin, and Cur-Art for 120 d	NUP 358 ranking ↑ CDK 5 expression in FA-stressed cells ↓	[138]
Stable cell line HEK 293-Tau 3R overexpressing Tau protein; SH-SY5Y cells; AREc32 cells; BV2 cells	5 and 10 μM (HEK 293-Tau 3R) or 100 μM (SH-SY5Y), incubated for 24 h	Nrf2–ARE pathway ↑ Aggregation of Tau PHF 6 peptide ↓ Inflammatory response ↓	
PD			
SH-SY5Y cells; BV-2 cells	0.1 to 20 μM CUR	CUR/HP-β-CD envelope showed significantly higher cellular uptake than CUR-CS-PLGA-NPs at 4 h CUR toxicity ↓ Oxidation ↓ Inflammatory response ↓	[120]
Rat adrenal pheochromocytoma cell line (PC12)	1 mg/mL MTT solution, 37 °C for 4 h	Cell damage ↓ Oxidative stress ↓ Expression of HO-1 and antioxidant enzymes such as SOD and CAT ↑ Cell membrane integrity ↑ Keap 1/Nrf2/HO-1 signaling pathway ↑ Apoptosis signaling pathway ↓	[139]
Hippocampus single-cell suspension; Wistar rats	Bulk curcumin at 5, 10, and 20 mg/kg, i.p.; Cur-PLGA-NPs at 5, 10, and 20 mg/kg, i.p., single daily	NSC proliferation and neuro-sphere formation ↑ Increased bioavailability of Cur-PLGA-NP ↑ Cell proliferation in hippocampus and SVZ ↑ Neuronal differentiation and neurogenicity genes ↑ Wnt/β-collagen signaling pathway ↑	[122]
		Phosphorylation of beta-conjugated proteins ↓ Cell cycle protein D1 ↑ TCF/LEF promoter ↑	
Mixed neurons/glial cells	10 μM curcumin	Spatial memory deficits ↓ Cholinergic neuron function ↑ Activation of microglia and astrocytes ↓ Cytokines ↓ Nuclear factor κ B (NF-κB) signaling pathway ↓ Neuroinflammation ↓ PPAR-γ ↑	[113]
SHSY5Y cells	1, 2.5, 5, 10, and 15 μM for 24 h	SUMO-1-JNK-Tau-shaft overactivation ↓	[140]
		Oxidative stress ↓	
Older adults aged 30–70 years; body mass index (BMI) 25–45 kg/m^2^	2 × 500 mg curcumin tablets providing 180 mg of curcumin per day	Circulating GSK-3β ↓ IAPP ↓ Insulin resistance ↓ Risk of T2D and AD ↓	[141]
SD rats (male, 7 weeks old, 180–200 g); C57BL/6 mice (8 weeks old, 22–26 g)	3 mg/kg, i.v., Fe-Cur NCP	Motor dysfunction ↓ Dopaminergic system and behavior ↑	[131]
Human macrophages, leukemia virus-transformed mouse macrophages, and SH-SY5Y cells	5, 10, 20, 40, and 80 μM treatment for 24 h	Oxidative stress ↓ Excess ROS ↓	[131]
SD rats	200 mg/kg, i.g.	Motor function deficits ↓ Denaturation of neuronal cells ↓	[127]
Musca domestica	0.037% final concentration (equivalent to 1 mM CUR)	ROS ↓ Motion defects ↓ Denaturation of DA ↓ Dopamine deficiency ↓ Climbing ability disorder ↓	[128]
SD rats (male, 7–9 weeks old, 350–400 g)	40 mg/kg, i.p., once daily for 21 days	Astrocyte activation ↓ NADPH oxidase complex ↓ NF_(IB) ↓	[142]
		TNF-α ↓ IL-1b and IL-1a ↓ iNOS ↓	
		Intrinsic apoptosis pathway (Bax, Bcl-2, caspase 3, and caspase 9) ↓ Glutathione system (GSH, GSSG, and redox ratio)↑ α-Syn aggregation ↓	
SH-SY5Y human cells	1 μM, 1 h before transfection	Mitochondrial ROS ↓ Caspase-3/7 ↓ PARP cleavage ↓ Neurodegeneration ↓	[143]
Albino rats (male, 3–6 months, 150–200 g)	30 mg/kg dissolved in DMSO daily for 60 days	Nystrom’s particles ↓ Purkinje cells and GFAP-γ-positive cells ↑ Baton twirling activities ↑ Acetylcholinesterase, glutathione, and superoxide dismutase ↓ Significant increase in malondialdehyde ↑	[124]
SH-SY5Y neuroblastoma cells	2 μM curcumin for 1 h	Cell viability ↑ ETS capacity ↑ Alternate respiratory capacity ↑	[144]
C57 BL/6 J wild-type mice (male, 8 weeks old, 15–20 g)	120 mg/kg, i.g.	Bioavailability ↑ Movement disorders ↓ Nigral DA degeneration ↓	[132]
C57 BL/6 mice (male, 25–30 g)	12, 24, and 48 mg/kg, CNB-0016	Behavioral disorders ↓ Corpus striatum dopamine, DOPAC, and HVA loss ↓ Accumulation of nitrite and citrulline ↓ Pro-inflammatory factor expression ↓ Glial cell activation ↓ Apoptosis ↓	[126]

Abbreviations: ICR: Institute of Cancer Research (USA); GSH: glutathione; CAT: catalase; SOD: superoxide dismutase; GPx: glutathione peroxidase; MDA: malondialdehyde; AChE: acetylcholinesterase; Gab-2: Grb2-associated binder; Bag 1: Bcl-2-associated athanogene 1; TGF-β: transforming growth factor; Ccnd 2: recombinant cyclin D1; TNF-α: tumor necrosis factor; PARP 1: poly ADP-ribose polymerase; APP/PS1: amyloid precursor protein and the human progeria gene 1; SLCP: solid lipid curcumin granules; PFC: prefrontal cortex; EC: entorhinal cortex; SC: superior colliculus; SNEDDS: solid self-nanoemulsifying drug delivery system; TLR4: Toll-XI-like receptor-XI 4; NF-κB: nuclear factor κ B; mTOR: mammalian target of rapamycin; NUP: nuclear pore protein; CDK: cyclin-dependent kinases; FA: fatty acid; Keap1: Kelch-like ECH-associated protein 1; Nrf2: nuclear factor erythroid 2-related factor 2; Cur-PLGA-NPs: curcumin-encapsulated nanoparticles; NCPs: nanoscale coordination polymers; DOPAC: dihydroxy-phenyl acetic acid.
